# Bovine Respiratory Disease in Veal Calves: Benefits Associated with Its Early Detection by Lung Ultrasonography and Its Prompt Treatment with a Single Dose of a Fixed Combination of Florfenicol and Meloxicam †

**DOI:** 10.3390/ani14233499

**Published:** 2024-12-04

**Authors:** Anastasia Lisuzzo, Damien Achard, Alessio Valenza, Barbara Contiero, Luca Cozza, Eliana Schiavon, Giacomo Catarin, Fabio Conte, Enrico Fiore

**Affiliations:** 1 Department of Animal Medicine, Production and Health, University of Padua, 35020 Legnaro, Italy; anastasia.lisuzzo@unipd.it (A.L.); barbara.contiero@unipd.it (B.C.); giacomo.catarin@unipd.it (G.C.); 2 Ceva Santé Animale, 33500 Libourne, France; damien.achard@ceva.com; 3 Ceva Animal Health S.p.A., 20127 Milan, Italy; alessio.valenza@ceva.com; 4 Independent Researcher, 31054 Possagno, Italy; cozzavet@gmail.com; 5 Istituto Zooprofilattico Sperimentale delle Venezie, 35020 Legnaro, Italy; eschiavon@izsvenezie.it; 6 National Veterinary Service, ULSS 3, 30174 Mestre, Italy; fabio.conte@aulss3.veneto.it

**Keywords:** BRD, treatment, ultrasonography, lung lesion, bronchopneumonia, veal calves

## Abstract

Lung ultrasonography can facilitate bovine respiratory disease (BRD) detection and can potentially improve treatment outcomes. In this study, ninety-six veal calves were followed weekly with clinical and lung ultrasound examinations during the production cycle. Thirty-six calves had clinical signs and abnormal lung ultrasound scans (TRT, n = 36) and were promptly treated with florfenicol and meloxicam. Healthy veal calves without clinical signs and lung lesions were enrolled in the control group (CTR, n = 48), while 12 calves were excluded by the study. The clinical (Wisconsin and California scores), ultrasound and lung lesion scores, total lung consolidation area, treatment rates (success, relapse, mortality), average daily gain (ADG), carcass quality, and gross lesions of lungs at slaughterhouse were monitored. Results showed 91.7% of treatments were performed 3–28 days after arrival. Lung lesions occurred five days before the peak of clinical scores. Following treatment, lungs healed within 5 days with high treatment success rates (97.1% success rate in 45 days and 94.9% overall success rate without relapse). Finally, TRT had similar gross lung lesion prevalence, ADG, and carcass quality to CTR. These results suggest that early detection of BRD followed by a prompt treatment can lead to several key benefits for the health and the growth performances of veal calves.

## Simple Summary

Bovine respiratory disease (BRD) is often not detected in time, mostly because of the lack of sensitivity of clinical examinations. This frequently leads to a delay in the treatment of the disease and an increased usage of antimicrobials. Lung ultrasonography examinations have recently emerged as an additional tool that can facilitate the detection of BRD and potentially improve the outcomes of its treatment. The aims of this study were to monitor the evolution of BRD by clinical and ultrasonographic assessments in veal calves during one production cycle and the healing process of lung lesions following a single administration of a fixed combination of florfenicol and meloxicam. Our findings revealed that most treatments were performed between 3 and 28 days after arrival. Moreover, lungs lesions were detected five days before clinical scores. The following treatment was able to reduce lung lesion within 5 days in almost all animals without relapse. Furthermore, no differences in growth and performance were evidenced between treated and healthy animals. These results suggested that a prompt treatment with a fixed combination of florfenicol and meloxicam can lead to several key benefits for the health of veal calves.

## 1. Introduction

Bovine respiratory disease (BRD) is a syndrome involving infectious agents (both viruses and bacteria), host immune response, and environmental factors. The viral pathogens associated with BRD can increase the susceptibility to secondary bacterial infections [1,2]. This syndrome may affect up to 61% of veal herds, causing decreased animal health and wellbeing, reduced growth and productivity, and increased economic losses [3].

The diagnosis in field and treatment decisions in the veal livestock are generally based on examination for BRD clinical signs: ocular discharge, nasal discharge, ear droop or head tilt, cough, abnormal respiration, and hyperthermia [4]. However, the use of clinical examination as the only tool to detect BRD frequently leads to misdiagnosis and delayed treatment decisions due to its low sensitivity and specificity (~60%): a non-negligible proportion of healthy animals are defined as ill and receives antimicrobial treatment, while a significant number of diseased animals are not detected and treated [1,5]. This situation is not in line with current animal welfare standards and antimicrobial stewardship in farm animals [6,7]. Ideally, antimicrobial treatment should be limited to calves with a confirmed active bacterial infection of the lower respiratory tract, but this necessitates detection tools with more accuracy than clinical examination [3,8].

Ultrasonography is a non-invasive, cost-effective, and portable method for investigating different structures in real time [1]. Specifically, lung ultrasonography shows greater sensitivity (77–94%) and specificity (74–100%) compared to clinical signs to perform BRD diagnosis [3,9]. This increased sensitivity and specificity may improve calf health by providing a more accurate diagnosis and avoiding unnecessary antimicrobial treatment [1]. Additionally, early BRD diagnosis is a key factor to improve the treatment success rate and reducing the risk of treatment failure. Lung ultrasonography can detect lung lesions in both clinical and subclinical animals allowing to differentiate between an upper or lower respiratory tract infection. In fact, lung lesions often develop before clinical signs and remain unidentified for different time periods [10,11]. In addition, timing and characteristics of disease resolution may present long-term influences on animals’ performance. However, this area is still poorly studied [10,12]. A periodic follow-up using lung ultrasonography could be helpful to assess lung lesion response to treatment and evolution [13].

In calves, treatment of acute BRD frequently relies on the combined use of an antibiotic and non-steroidal inflammatory drug (NSAID). Among the various antibiotics available for this condition, florfenicol is often selected considering its activity spectrum that includes sensitive *Pasteurellaceae* and *Mycoplasmopsis bovis* (*M. bovis*; former genus name *Mycoplasma bovis*). Furthermore, the use of NSAID such as meloxicam may reduce lung tissue damages caused by lung inflammation which occurs concurrently to bacterial colonization of lungs [10,14,15]. Although a fixed combination of florfenicol and meloxicam has previously shown to be efficacious for the treatment of BRD under both field and experimental conditions [14,16], lung ultrasonography has never been used to document the evolution of lung lesions during and after treatment with this combination in calves with BRD.

For these reasons, the aims of this study were to monitor the evolution of BRD by clinical and ultrasonographic assessments in veal calves during one production cycle and the healing process of lung lesions following a single administration of a fixed combination of florfenicol and meloxicam.

## 2. Materials and Methods

### 2.1. Ethical Statement

Animal care and procedures are in accordance with the Guide for the Care and Use of Laboratory Animals and Directive 2010/63/EU for animal experiments (National law: D.L. 26/2014). No invasive medical procedures were executed to perform the study. The study was performed with the consent of the animals’ owner during the routinely extramural clinical activity of the Veterinary Teaching Hospital, University of Padua.

### 2.2. Animals

A single batch of 96 calves (group of calves that arrived at the farm at the same time to be fattened and slaughtered together) from a single farm located in Veneto region (Italy) was used for this longitudinal study. Animals originated from nine calf collection centers before the arrival on farm (A, n = 3; B, n = 9; C, n = 9; D, n = 42; E, n = 21; F, n = 3; G, n = 1; H, n = 3; and I, n = 5). The average weight and age of calves at arrival were 53 ± 5 kg and 30 ± 9 days, respectively. Four different breeds were represented (Holstein–Friesian, n = 4; Italian Simmental, n = 31; Rendena, n = 3; and Holstein–Friesian x Belgian Blue crossbreed, n = 58) as well as both genders (M = 84; F = 12).

Animals were housed in indoor pens (6 to 8 animals/pen) with a slatted floor. All calves had 1.5 to 1.8 m^2^/animals in accordance with European legislation (European Council Directive 2008/119/EC). Milk replacer (2 to 7 L/day/animal) was provided twice a day, and 50 g to 3.5 kg/day/animal of solid fibrous feed was provided after the morning meal of milk replacer, still according to European legislation.

Animals were monitored through clinical examinations and lung ultrasound evaluations during the production cycle (7 January 2023 to 7 July 2023) by veterinarians of the Veterinary Teaching Hospital of the University of Padua (Italy). The last clinical and lung ultrasound evaluations were between 3 and 7 days before the slaughterhouse.

### 2.3. Clinical Examination

Clinical examinations included the assessment of rectal temperature, cough, breathing pattern, nasal discharge, eye discharge, and ear position according to [17]. These clinical signs were used to calculate the California scoring system and the Wisconsin scoring system (Table S1). According to these clinical scores, animals were considered to have BRD if the California score ≥ 5 or if the Wisconsin score ≥ 4 [17,18].

### 2.4. Lung Ultrasonography Evaluation

Ultrasound evaluations were based on bilateral lung ultrasonography (LUS) in 6 regions based on intercostal space (ICS) scans: caudal region, from 10th to 7th ICS; middle region from 6th to 5th ICS; and cranial region from 4th to 3rd ICS, both in right and left thorax. All ultrasounds were scanned using a portable ultrasound scanner (Dramiński^®^ Ultrasound Scanner Blue, Dramiński^®^ S.A., Olsztyn, Poland) and multifrequency linear probe (6.0–15.0 MHz; Draminski^®^ S.A., Olsztyn, Poland). Animals were not clipped, and ethyl alcohol (90%) was used as a transducing agent. All scans were performed by a trained veterinarian regarding clinical examinations and ultrasonographic scans using a constant setting using a frequency of 6.0 MHz, 15 cm depth acoustics window, 100% gray scale gain. Time-gain compensation was in a neutral position. Images were saved in JPEG format. The saved ultrasound images were used for a post sampling measurement of the area (cm^2^) and thickness (cm) of consolidations using Image-J software ver. 1.54k (Wayne Rasband, Rockville Pike, Bethesda, MD, USA). The sum of consolidation area in the six regions provided the total area of consolidation (cm^2^).

**Figure 1:**
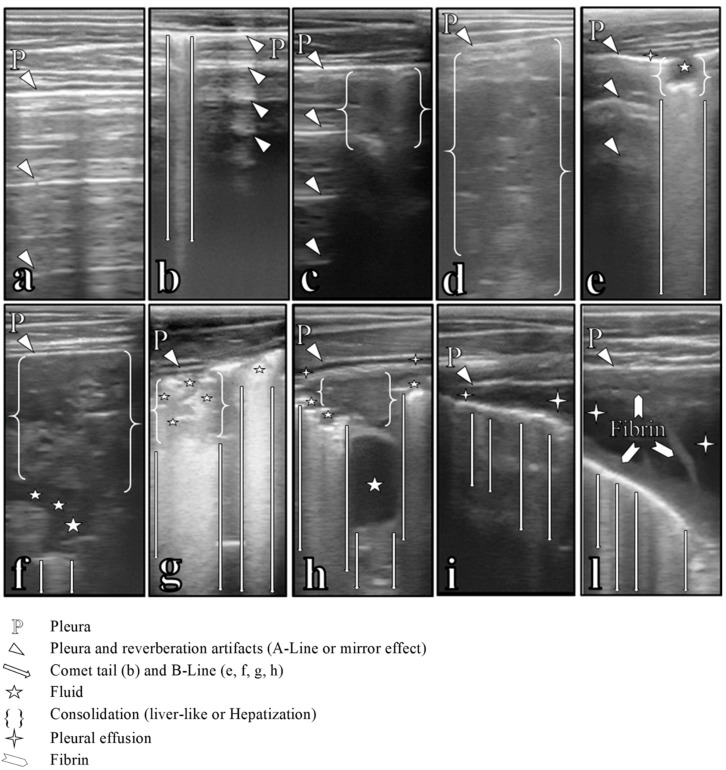
Main ultrasonographic lesions found in lung scans in animals enrolled in the trial. (**a**) Healthy lung; (**b**) comet tail; (**c**) lobular or spot liver-like or hepatization; (**d**) lobar liver-like or hepatization; (**e**) fluid alveologram with B-line and focal pleural effusion; (**f**) lobar liver-like or hepatization and fluid bronchogram with a small B-line; (**g**) lobar liver-like or hepatization and fluid alveolograms/bronchograms with B-lines; (**h**) lobar liver-like or hepatization, fluid bronchograms with B-lines and mild pleural effusion; (**i**) moderate pleurisy comet tails; and (**l**) severe pleurisy with fibrin layers and septa and comet tails.

Bilateral ultrasound evaluation of the lung was performed to establish two different scores: the ultrasonography score (US) and the lung lesion score (LLS). The US involves a 6-point scale [9]: 0—healthy lung; 1—presence of comet tails; 2—spot of lobular lung consolidation; 3—lobar lung consolidation of one lobe; 4—lobar lung consolidation on two lobes; 5—lobar lung consolidation on at least three lobes. Abnormal lung consolidations were characterized as a score ≥ 3. The LLS was based on a previous study in [1] that evaluates the types of lesions found in the six ultrasonography regions (cranial, middle, and caudal of both lungs) and was slightly modified as follows: 0—healthy lung; 1—presence of comet tails; 2—spot of lobular consolidation; 3—lobar consolidation; 4—lobar consolidation and comet tails; 5—fluid alveologram/bronchogram; 6—fluid alveologram/bronchogram and comet tails; 8—lobar consolidation and fluid alveologram/bronchogram; 9—lobar consolidation, fluid alveologram/bronchogram, and comet tails; and 11—pleuritis (Figure 1). The score for each region established as previously indicated was summed to calculate the LLS. Animals were considered as affected by BRD if the LLS was greater than 10.5 [19]. In addition, a rough assessment of the lesions thickness in cranial regions was performed during the ultrasound itself using the graded scale of ultrasound scanner. This assessment was used to discriminate lesions in cranial regions using the 3 cm threshold (suspected active bronchopneumonia ≥ 3 cm) [20].

### 2.5. Treatment, Group Division, and Necropsy Examination

Animals with a lung lesion in the cranial lobe ≥ 3 cm or US ≥ 3 and at least one clinical sign compatible with BRD (>39.4 °C, cough, altered breathing, nasal discharge, eye discharge, or ear position) were included in the treated group (TRT group). Animals enrolled in this group were sampled with a deep nasopharyngeal swab equipped with sheat (35 cm; Medical Wire and Equipment Co., Ltd., Corsham, UK) to perform bacteriological (swabs placed in blister pack) and virologic (dry-swab) analysis and antibiograms (MICs) to assess the sensitivity to florfenicol, amoxicillin and clavulanic acid, ampicillin, ceftiofur, enrofloxacin, flumequine, gamithromycin, spectinomycin, tildipirosin, tilmicosin, trimethoprim, sulfonamides, tiamulin, tulathromycin, kanamycin, and tetracycline. Immediately after, a single subcutaneous administration of a fixed combination of florfenicol and meloxicam was given to calves (400 mg/mL florfenicol and 5 mg/mL meloxicam; SC 1 mL/10 kg BW; Zeleris, Ceva Salute Animale S.p.A., Milan, Italy).

Animals without the above-mentioned treatment criteria and that did not receive any kind of treatment during the production cycle were included in the control group (CTR group), while the remaining animals were excluded by the study.

The success (animals that improved after treatment), relapse (animals that required retreatment), chronicity (animals without improvements and with more than three treatments), and mortality (dead animals for BRD) rates of the treatment were evaluated until 45 days after treatment (45-day rates) [21] and for all the production cycle (overall rates). After 45 days from the first treatment, it was considered as a second BRD case.

At the end of the production cycle, animal weight before slaughtering and cold carcass weight were collected. These data were used to calculate the average daily gain (ADG; kg/day). The lungs of all animals were collected at the slaughterhouse for necropsy examination, as well as animals that died during the production cycle. The type of lung lesions was recorded, and a classification of healthy (Score 0) and pathological (Score 1) lungs was determined by veterinarian of the Italian national veterinary service.

### 2.6. Scheduling of Clinical and Ultrasound Evaluations

All animals received clinical and ultrasound evaluation at least once a week. In addition, animals that were progressively enrolled in the TRT group received additional evaluations at +1, +3, +5, +7, +9, +11, and +14 days after treatment. Thereafter, these animals still continued clinical and ultrasound evaluations at least weekly until the end of the production cycle.

### 2.7. Statistical Analysis

Statistical analysis was conducted with S.A.S. system software (version 9.4; SAS Institute Inc., Cary, NC, USA). The parameters under evaluation (Wisconsin score, California score, US, LLS, total area of consolidation, thickness of consolidation, thickness of pleuritis) presented a non-normal distribution at the Shapiro–Wilk test. Instead, the distribution of these data followed a Poisson distribution.

Considering that the treatments were performed over several days of the production cycle, the comparisons between TRT and CTR groups were performed the day of arrival of animals, the day of treatment for each treated animal, and the day before slaughter for each animal. Moreover, the statistical model of the follow-ups was set retrospectively considering also the examinations carried out before diagnosis and treatment, during all the production cycle (180 days), the percentage of sick animals in different months of the year, and the number of evaluations.

The comparison between CTR and TRT groups at standard time points and the follow-ups of the TRT group were analyzed by a generalized linear mixed model with Poisson distribution (PROC GLIMMIX) procedure. The dependent variables in the comparison were clinical scores (Wisconsin and California), ultrasound scores (US and LLS), and total lung consolidation area. This mixed model included the fixed effect of group, time, breed, gender and their interactions, the random and repeated effect of the animal, and the random effect of the collection center. Furthermore, age at arrival was considered as covariate. The dependent variables in the follow-up model included clinical scores, ultrasound scores, total lung consolidation area, consolidation area and thickness for cranial and middle regions. This mixed model used the same fixed effects, random effect, and covariate with the exception of the fixed effect of group (analysis limited to the follow-up of TRT group). The hypotheses of both models on the residuals were graphically assessed.

The comparison between groups in animal’s growth and performance was evaluated by a one-way ANOVA considering the group effect, whereas the carcass quality (S-EUROP classification; [22]) and lung lesions at the slaughter house were evaluated with a Chi-square test by the MedCalc software ver. 23.0.9 (MedCalc Software, Ostend, Belgium). The latter analysis was also used to assess differences in diseased animals according to clinical scores and in clinical signs between groups and over time.

In all models, post hoc pairwise comparisons among least squares means were performed using Bonferroni correction. The *p*-value accepted as significant was ≤0.05.

The diagnostic test evaluation (MedCalc software for Windows ver. 19.4, Ostend, Belgium) was assessed for the California score (affected by BRD if the score was ≥5), Wisconsin score (affected by BRD if the score was ≥4), US (affected by BRD if the score was ≥3), and LLS (affected by BRD if the score was ≥10.5) compared to necropsy findings (gold standard test). The last examination between 3 and 7 days before the slaughterhouse was used for this evaluation. Furthermore, presence of pleural adhesion was considered as previous cases of pleuritis [23] recorded by the LLS score.

## 3. Results

### 3.1. General Results of the Treated Group

Forty-seven animals showed clinical signs indicative of BRD. Among them, 11 animals had no lung lesions evidenced by lung ultrasonography, and therefore they were categorized as upper respiratory tract (URT) disease (presence of clinical signs without lung lesions) and excluded from statistical analysis. The remaining 36 animals were then enrolled in the TRT group (TRT, n = 36). The other forty-nine animals did not show clinical signs of BRD and did not present lung lesions during ultrasonography investigation. Of these animals, one calf received treatment due to ruminal meteorism, and it was excluded from the study. Consequently, the CTR group enrolled 48 animals that never received treatment (CTR, n = 48).

**Figure 2:**
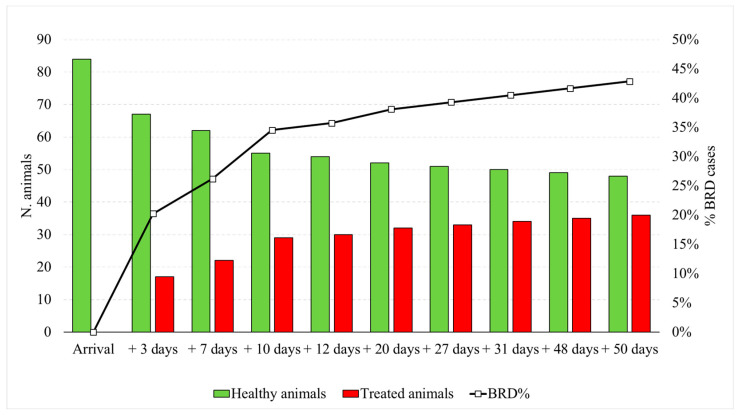
Number of animals enrolled and progressively treated (control group—CTR, n = 48; treated group—TRT, n = 36) represented by histograms and left *y*-axis, and cumulative percentage of bovine respiratory disease (BRD) cases represented by line and right *y*-axis.

A total of 33 (91.7%) and 3 (8.3%) animals were enrolled in the TRT group, respectively, during the first 4 weeks and during 5–8 weeks after the arrival. In particular, animals were enrolled as follows (Figure 2): +3 days after arrival (n = 17; 47.2%); +7 days after arrival (n = 5; 13.9%); +10 days after arrival (n = 7; 19.4%); +12 days after arrival (n = 1; 2.8%); +20 days after arrival (n = 2; 5.6%); +27 days after arrival (n = 1; 2.8%); +31 days after arrival (n = 1; 2.8%); +48 days after arrival (n = 1; 2.8%); and +50 days after arrival (n = 1; 2.8%).

The bacteriological and virologic analysis revealed the presence of coronavirus (32%; n. animals: 16), *M. bovis* (18%; n. animals: 9), *Pasteurella multocida* (46%; n. animals with only *P. multocida*: 6; n. mixed infection with *T. pyogenes*: 1; n. mixed infection with Coronavirus: 16), and *Trueperella pyogenes* (4%; n. animals: 1) in the nasal swabs of the BRD calves. Coronavirus was always identified in a co-infection with bacteria, while only one case had a co-infection of *P. multocida* and *T. pyogenes*. All bacterial strains that were tested for antimicrobial susceptibility were sensitive to florfenicol; resistant strains of *P. multocida* against kanamycin, tetracycline, spectinomycin were found in three animals.

A total of three animals of the TRT group died during the study period, and a field necropsy examination with tissue sampling for bacteriological examination was performed. The first animal died from a multi-resistant *E. coli* infection at +7 days after arrival and +4 days after BRD treatment. A second calf died for severe pleural effusion at +109 days after arrival and +106 days after treatment. Post mortem bacteriological analysis on lungs and pleural effusion fluid revealed the presence of *Mannhemia haemolytica* and *hemolytic E. coli*. The last animal died at the end of the production cycle from cecocolic torsion (+171 days after arrival and +122 days after treatment). Consequently, only one animal died for the BRD case.

Additionally, one animal had chronic lung lesions until slaughtered confirmed with lung gross examination. Moreover, three animals of the TRT group showed a second BRD case and were treated with the fixed combination of florfenicol and meloxicam. One animal was treated +50 days after the first treatment (+60 days after arrival), and other two animals were treated after +69 days (+72 days after arrival).

Considering the first 45 days after treatment, the success 45-day-rate was 97.1% (n. 34/35) and the chronicity 45-day-rate was 2.9% (n. 1/35) excluding the dead animal for *E. coli*. No animal required a second treatment (relapse 45-day-rate 0%) as well as no animal died for BRD (mortality 45-day-rate 0%) during this time frame. At the end of cycle, the overall success rate was 94.9% (n. 36/38), the overall relapse rate was 0%, the overall chronicity rate was 2.6% (n. 1/38), and the overall mortality rate was 2.6% (n. 1/38) considering the three second BRD cases.

### 3.2. Clinical and Ultrasonographic Results in Treated and Control Groups

At arrival, no differences were observed in Wisconsin score, California score, US, LLS, and total lung consolidation area between calves in the TRT and CTR groups (Table 1). Disease prevalence at arrival was very low for Wisconsin clinical score (2.1% in CTR, and 0% in TRT groups; *p* = 0.385) and California clinical score (6.3% in CTR, and 2.7% in TRT groups; *p* = 0.446).

At treatment day, the calves in the TRT group showed higher clinical (Wisconsin and California scores) and ultrasonographic (US, LLS, and total lung consolidation area) evaluations compared to the calves in the CTR group. However, the higher clinical scores observed in the TRT group were generally not indicative of BRD. In fact, disease prevalence remained low for Wisconsin clinical score (6.3% in CTR, and 13.8% in TRT groups; *p* = 0.249 between groups; *p* = 0.308 CTR over time, and *p* = 0.022 TRT over time) and California clinical score (8.3% in CTR, and 25% in TRT groups; *p* = 0.037 between groups; *p* = 0.708 CTR over time, and *p* = 0.007 TRT over time). At the end of the production cycle, clinical scores were low and very similar to the scores obtained at arrival in both groups. On the contrary, the US, LLS, and total lung consolidation area were all above the scores observed at arrival in both TRT and CTR groups. The US and LLS were greater in the TRT group compared to the CTR group at that time, but these parameters were not elevated enough to qualify for BRD (US < 3; and LLS < 10.5).

Regarding the pathological findings evidenced at the slaughterhouse or during necropsy examinations during the production cycle, 23.8% (n = 20; 19 from slaughterhouse and 1 from necropsy examination) of the calves enrolled had lung lesions, 20.8% (n = 10; all from slaughterhouse) in the CTR group, and 27.7% (n = 10; 9 from slaughterhouse and 1 from necropsy examination) in the TRT group (*p* = 0.687). No differences were observed between the two groups in ADG, live weight and cold carcass weight at the end of the production cycle (data for dead animals not available) as well as in carcass quality according to S-EUROP classification (Table 2).

### 3.3. Follow-Up of the Treated Group

**Figure 3:**
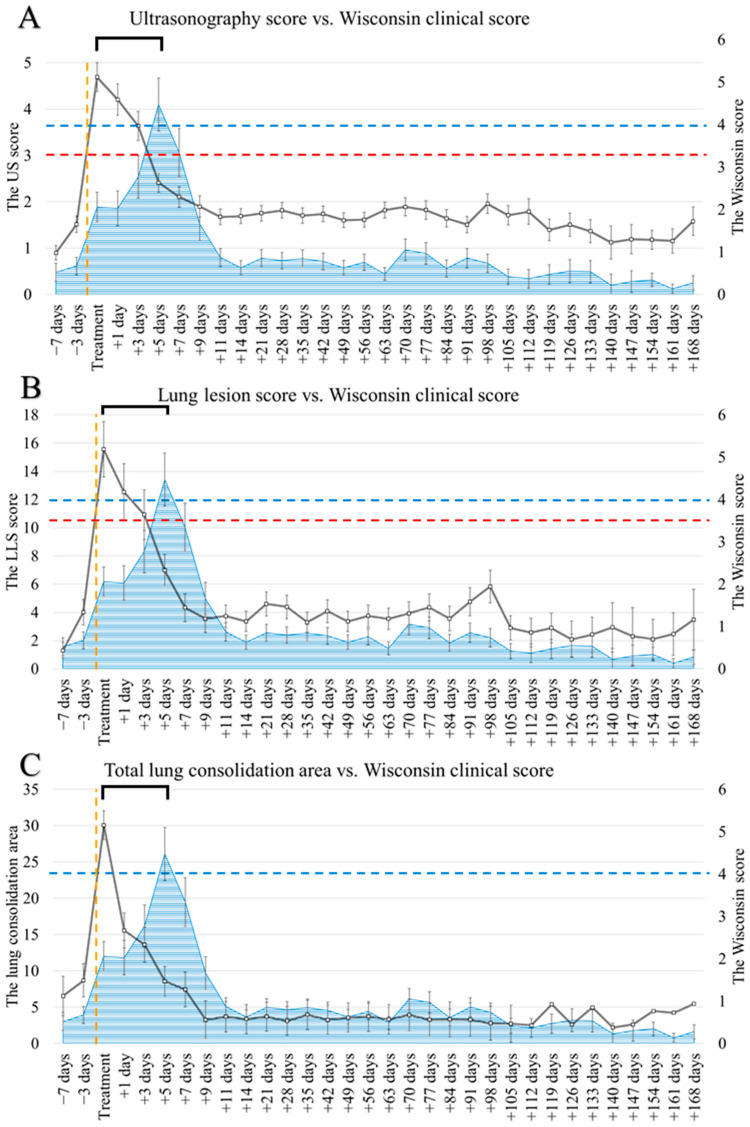
Comparison over time in treated animals (TRT, n = 36) using mean values and SEM: (**A**) ultrasonography score (US) vs. Wisconsin score; (**B**) lung lesion score (LLS) vs. Wisconsin score; (**C**) total lung consolidation area (cm^2^) vs. Wisconsin score. The dashed orange lines were used to evidence the day of diagnosis and treatment. The continued black lines were used for lung ultrasound evaluation (US, LLS, and total lung consolidation area), while the dashed red lines were used to evidence the cut-offs of US and LLS scores. The blue areas and dashed lines evidenced the Wisconsin clinical score with its cut-off value. The black square brackets were used to evidence the 5 days of difference between the peak for lung ultrasonography (US, LLS, and total lung consolidation area) and the peak for clinical scores (Wisconsin).

Both clinical scores (Wisconsin and California), US, LLS, and the total area of consolidation showed significant changes over time in the follow-up of the TRT group (Figure 3). The means, medians, and statistical differences can be consulted in Table S2.

The clinical scores, Wisconsin and California, showed the highest values at +5 days after treatment preceded by a significant increase on the day of diagnosis and treatment. Only five (13.8%) and nine (25%) animals showed a Wisconsin and California score, respectively, indicative of disease at the day of diagnosis, compared to thirty-one (91.2%) for both clinical scores at +5 days (*p* < 0.001). The clinical signs presented by calves on the day of diagnosis were, in decreasing order of frequency, and vs. +5 days: cough (41.7% vs. 76.5%, *p* = 0.003), nasal discharge (25.0% vs. 85.3%, *p* < 0.001), ear droop or head tilt (16.7% vs. 14.7%, *p* = 0.820), body temperature above 39.4 °C (13.9% vs. 2.9%, *p* = 0.103), ocular discharge (5.6% vs. 32.4%, *p* = 0.004), and abnormal breathing (0% vs. 0%, *p* = 0.999). After the peak at +5 days, the clinical scores decreased significantly within 2–4 days (+7 to +9 days after treatment).

**Figure 4:**
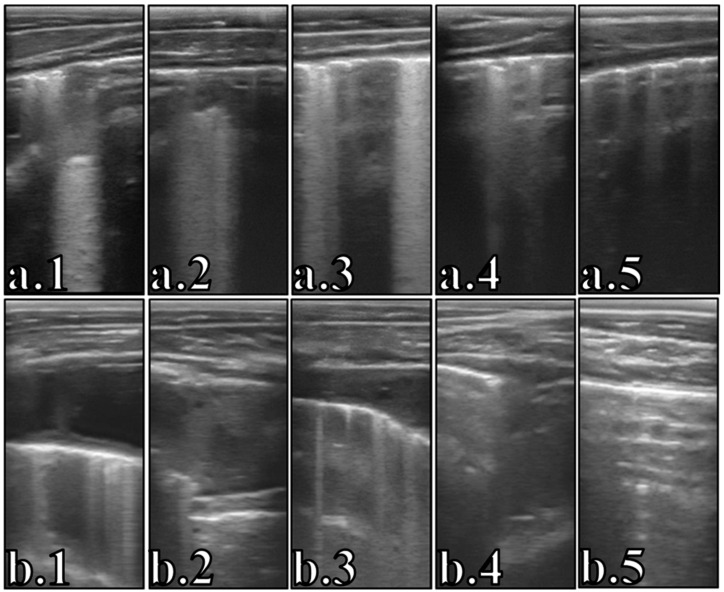
Examples of healing evolution processes of lobular lesion liver-like or hepatization and fluid bronchogram with B-line (**a.1**–**a.5**) and severe pleurisy with fibrinous layers and septa and comet tails (**b.1**–**b.5**). (**a.1**) Day of diagnosis and treatment; (**a.2**) +1 day after treatment; (**a.3**) +5 days after treatment; (**a.4**) +7 days after treatment; (**a.5**) +21 days after treatment. (**b.1**) Day of diagnosis and treatment; (**b.2**) +3 days after treatment; (**b.3**) +5 days after treatment; (**b.4**) +7 days after treatment; (**b.5**) +21 days after treatment.

Both US and LLS showed the greatest values the day of diagnosis and treatment (US: 4.69, LLS: 15.56). Afterwards, the US score reduced significantly at +3 days (score: 3.64), +5 days (score: 2.41), and +11 days (score: 1.68) after treatment. This score reduced under the threshold indicative of lung bronchopneumonia (US ≥ 3) at +5 days after treatment and remained almost similar during the study period following the +11 days. The LLS decreased significantly at +1 day, +5 days, and +7 days after treatment. As for the US, the LLS reduced under the threshold of BRD (LLS ≥ 10.5) at +5 days after treatment and the trend of the values following the +7 days were similar during the study period. The total area of consolidation was highest at the day of diagnosis and treatment (30.06 cm^2^) followed by a significant decrease at +1 day (total area: 15.56 cm^2^), +5 days (total area: 8.57 cm^2^), and +9 days (total area: 3.26 cm^2^) after treatment (Figure 4). After the +9 days from treatment, no significant changes were recorded in consolidation areas.

Both consolidation areas and thickness of the cranial regions showed a peak the day of diagnosis and treatment with an area of 13.1 and 15.6 cm^2^ and a thickness of 5.0 and 4.5 cm regarding the right and left regions, respectively. The consolidation areas reduced significantly at +1 day and +9 days for the right region, and at +1 day and +5 days for the left region. Instead, the consolidation thickness reduced significantly at +3 days, +5 days, and +11 days for the right region, and at +3 days and +5 days for the left region (Table 3). Regarding the consolidation areas of the middle regions (Table S3), the lesions reduced significantly at +5 days and +1 day for the right and left regions, respectively, after a peak the day of diagnosis and treatment.

Among the 36 animals of the TRT group, a total of 28 animals showed at least one case of pleuritis with pleural effusion during the trial. Pleural effusion increased the day of diagnosis and the day after followed by a significant decrease at +1 day for the middle right region, +3 days for the cranial right and middle left regions, and +5 days for the cranial left region (Table 4).

### 3.4. Diagnostic Test Evaluation

**Figure 5:**
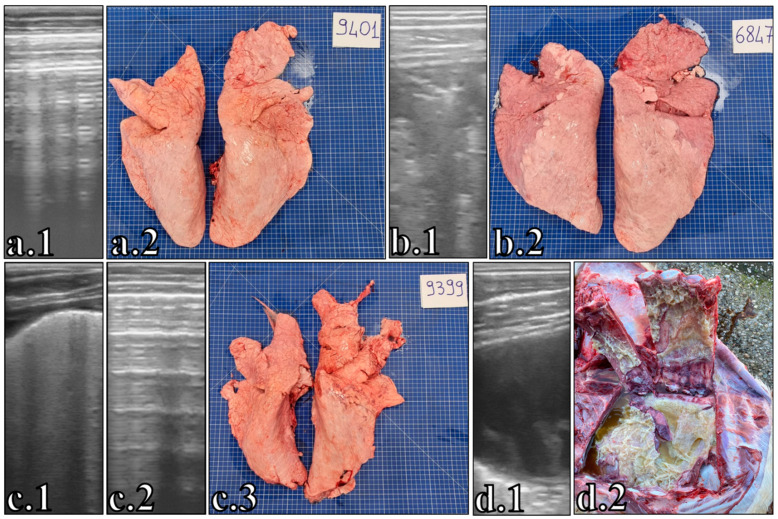
Example of the comparison between lung ultrasonography and lung gross lesion in four animals (**a**–**d**). (**a.1**) Lung ultrasonography 3 days before slaughtering indicative of healthy lung; (**a.2**) healthy lungs at slaughter house; (**b.1**) last lung ultrasonography of the only chronic animal 3 days before slaughtering indicative of lobar liver-like or hepatization; (**b.2**) atelectasis of cranial lobes at slaughter house; (**c.1**) lung ultrasonography on the day of diagnosis and treatment indicative of moderate pleurisy with B-lines; (**c.2**) last lung ultrasonography 3 days before slaughtering indicative of healthy lung; (**c.3**) lung with pleural adhesion in cranial lobes indicative of past episodes of pleurisy at slaughter house; (**d.1**) last lung ultrasonography on the day of death indicative of severe fibrinous pleurisy with fibrin layers and septa; (**d.2**) necropsy findings of thoracic cavity immediately after death.

Considering the comparison between necropsy findings and the last clinical and ultrasound examination (3 to 7 days before the slaughterhouse), the accuracy of the Wisconsin score, California score, and US scores were less than 80%, while the LLS showed an accuracy of around 93%, indicating excellent accuracy (Table 5). Both clinical scores showed excellent specificity (100%) but very low sensitivity (<8%). In contrast, both ultrasound scores (US and LLS) showed lower specificity compared to clinical scores (90% and 96%, respectively) but greater sensitivity (48% and 85%, respectively). Moreover, the LLS showed greater sensitivity and specificity compared to the US (Figure 5).

## 4. Discussion

Animal management by producers and veterinarians represents an important key point in preventing and reducing BRD cases on a farm. Therefore, the knowledge about specific BRD outbreak patterns in each farm should be investigated [21]. In fact, the timing of BRD onset and consequent peak appears really variable: 3 days after arrival, or after 1, 2, 3, or 6 weeks [6,11,24]. Thus, the time between animals’ arrival and first treatment is generally between 2 and 8 weeks [21]. Furthermore, BRD may affect from 7% to 61% of the herd resulting in reduced animals’ health and welfare, as well as important economic losses [3,11]. The results of this study were in accordance with the literature, presenting the BRD onset associated with the first treatment at +3 days after arrival to +50 days (around 92% of BRD cases in the first 4 weeks) and a cumulative prevalence of about 43%. These findings confirmed that a close monitoring of animal health should be provided in the first month after arrival in order to perform an early diagnosis and treatment.

*M. bovis*, *Pasteurella multocida*, *Mannhemia haemolytica*, and *Trueperella pyogenes* are the most commonly isolated bacteria in animals affected by BRD [6,24]. In this study, a greater predominance of *P. multocida* (45.5%) followed by *M. bovis* (18.2%) was found, confirming the importance of controlling these two bacteria within the herd. Bovine coronavirus was found in about 32% of isolated pathogens in this study, always in association with other bacteria. In fact, this virus may be an important risk factor when associated with other microorganisms as a predisposing factor [12,25].

Regarding treatment efficacy, a success rate of 80–85% after one treatment is usually expected [26]. In this study, the success rates measured were higher with 97.1% in the first 45 days and 94.9% in all the production cycle (180 days). The BRD mortality usually ranges from 1.5 to 6% [11,24,27]. Only a single calf died for BRD in this study (2.6%), in agreement with the literature. This fatal outcome of BRD occurred +106 days after treatment, possibly following a second clinical episode of BRD in the last tier of the production cycle. A normal relapse rate is about 20–35% [28]. In our study, no case of relapse was identified. A second BRD case was observed for three calves as suggested by the increase in lung lesions at +50 days and +69 days from first treatment. Furthermore, these events were simultaneous with the introduction of a new batch of younger calves into the barn, which probably led to a new circulation of pathogens.

Pulmonary infections and subsequent lung consolidations may occur early in life [2]. Consequently, both veal calves and fattening bulls at arrival may already show lung consolidation [1,6]. However, it must be considered that these areas may indicate an active bronchopneumonia, with inflammation and infection requiring treatment, or inactive bronchopneumonia, where both inflammation and infection are no longer present [3,8]. In our study, there were no differences between groups according to clinical scores (Wisconsin and California), ultrasound scores (US and LLS), and total lung consolidation area on calves’ arrival. The latter parameter indicated the presence of small areas of lobular consolidation in some animals. Specifically, only the 16.7% and 5.6% of the CTR and TRT groups, respectively, showed a consolidation > 1 cm in the cranial lobes on arrival and without statistical differences between groups. This finding may suggest a limited presence of active bronchopneumonia on arrival, which could have positively influenced the high treatment efficacy. Also, an early BRD detection may have contributed to the excellent treatment success rate. In fact, classical scoring systems such as Wisconsin or California were only abnormal in a small proportion of calves at the time of treatment (14–25%) while they confirmed sickness in nearly all calves in the TRT group (92%) 5 days later. Consequently, results from our study suggest that the case definition used to detect BRD (US ≥ 3 and at least one BRD clinical sign) was useful to initiate treatment very early in the disease course.

At the end of the production cycle (180 days), there were no differences in clinical scores between groups. In contrast, ultrasound scores (US and LLS) were higher in the TRT group. However, these scores were lower than the threshold value for disease. In fact, the US score had a mean value indicative of lobular consolidation (US = 2). As previously discussed, the evaluation of consolidations should be performed carefully as this may be an active bronchopneumonia, which can therefore progress over time involving more parenchyma, or an inactive BRD, i.e., undergoing resolution post infection, scar tissue, or chronic lesions. This distinction can be facilitated by performing multiple lung ultrasound evaluations over time [1,3]. The animals in this study were evaluated at least weekly, so these lobular consolidations remained stable throughout the production cycle (inactive BRD).

The prevalence of lung lesions at the slaughterhouse are reported to be 41 to 90% [11,29]. In this study, a much lower prevalence of lung lesions was found in both CTR (20.8%) and TRT (27.7%) groups (overall prevalence 23.8%, n = 20). This could be a beneficial outcome arising from the weekly monitoring program established for the trial enabling early detection and treatment of sick calves. Usually, lung ultrasonography displays a good association with lung gross lesion [10]. However, it should also be considered that the consolidations observed with lung ultrasonography can be either an indication of viral or bacterial infection (both active bronchopneumonia), but also indicative of inactive bronchopneumonia [8,10]. Consequently, antimicrobial treatments targeting bacteria might not be effective in cases of viral or chronic pneumonia, or in the absence of infection. In fact, different antimicrobial protocols showed absence of effect on the lung lesions at slaughter as opposed to anti-inflammatory treatments that had a positive effect [10,12,29]. The findings of this study suggested that targeted treatments following periodic clinical and ultrasonographic monitoring allowed an early diagnosis of active bronchopneumonia with consequent reduction in the prevalence of lung lesions at slaughter.

BRD is associated with reduced animal growth and productivity, and increased levels of lung lesions identified by ultrasonography are similarly negatively associated [12,28,30]. Lower ADG might be a consequence of reduced food intake and increased requirement in protein and energy for inflammatory and immune responses during disease status [6]. In our study, CTR and TRT groups did not differ relatively to calf ADG and carcass quality (S-EUROP conformation, and fat score 1–5). It should be considered that the enrollment in the TRT group began 3 days after arrival and ended during the first 50 days of the production cycle (180 days). Therefore, it is possible that early diagnosis and treatment were effective in resolving the BRD outbreak by allowing the animals to recover the possible reduced productivity in the following 130 to 177 days of the cycle [3,19].

Regarding the follow-up of the TRT group, the clinical scores increased on the day of diagnosis and treatment, but the peak was realized at +5 days after treatment. Thereafter, these scores decreased within 2–4 days (+7 to +9 days from treatment). Clinical signs may be delayed by seven days post induced viral infection, or one to four days after induced bacterial infection [10,12]. According to study [31] on young bulls, hyperthermia occurred up to 12 h before the BRD clinical signs: nasal discharge (24 h), depression (51 h), cough (65 h) and eye discharge (80 h). Effectively, the immune response following infection exhibits a cellular and fibrin clearance that may begin 1–2 days, up to 7–10 days, after disease onset [28]. The findings of this study agree with the literature: cough, nasal, and ocular discharges increased from the day of diagnosis and treatment up to +5 days later. Moreover, their reduction in the following 2–4 days (+7 to +9 days from treatment) was in accordance with the typical evolution and clinical resolution of BRD [12].

Early BRD diagnosis is a key factor in improving the success rate and reducing the risk of treatment failure. Lung ultrasonography can be a valuable screening tool for animals due to its ability to identify cases with or without an involvement of the lung parenchyma despite the clinical presentation [10,11]. In fact, lung lesions often develop before clinical signs, as also previously discussed, and remain unidentified for longer or shorter periods [11]. Furthermore, lung ultrasonography can assess different types and severity of lung lesions that may affect treatment choices [1,32]. The current indication for the treatment of BRD includes two categories: lower versus upper airway infection and depth/thickness of lesions. In the former case, the recommendation is to treat with antimicrobials only the lower respiratory tract infections. Additionally, treatment is recommended when it is identified an active bronchopneumonia (both infection and inflammation present) having a depth/thickness ≥ 3 cm [3,8]. In this study, animals were followed up at least weekly with lung ultrasonography, thus allowing probable better identification of an active bronchopneumonia status [1]. In addition, animals had a peak in US score (4.69; cut-off value = 3), LLS score (15.56; cut-off value = 10.5), total lung consolidation area (30.1 cm^2^), areas of consolidation (13.1 and 15.6 cm^2^ for right and left lung, respectively), and lesion thickness (5.0 and 4.5 cm for right and left lung, respectively) of the cranial regions on the day of treatment. These results suggest that a systematic lung ultrasonography allows for an early BRD diagnosis with active bronchopneumonia 5 days earlier than clinical signs in this study. The early diagnosis and treatment might therefore have positively influenced the high success rate (97.1% as a 45-days-rate and 94.9% as overall rate) without relapse rate (0% as a 45-days-rate and overall rate vs. 25–30%) and reduced lung lesions at the slaughter (23.8 vs. 41–90%).

The duration of resolution following acute case of BRD is a topic of recent interest for which lung ultrasonography is acknowledged to be useful [10]. Indeed, the evolution of ultrasound scores and lung lesions allows the evaluation of the process of animal recovery and tissue healing [1,12]. Moreover, lesion regression appears to be highly sensitive in predicting the resolution of the inflammatory and infectious process [33,34]. However, periodic follow-up is necessary to perform this assessment in response to treatments [13]. After treatment with florfenicol and meloxicam, the US score decreased after +3 days, +5 days, and +11 days. Similarly, LLS showed improvement after only 1 day, +5 days, and +7 days after treatment. In addition, animals were no longer considered sick (US < 3 and LLS < 10.5) from +5 days after treatment. The difference in reduction times of the two scores (US and LLS) may be due to the type of lung lesions considered: in the former case, they were only comet-tail and lung consolidation, whereas in the latter, they were comet-tail, hepatization (liver-like), fluid alveolograms/bronchograms, and pleurisy [1,9]. Compared to LLS, the total lung consolidation area was reduced as early as the day after treatment (+1 day), +5 days, and +9 days after treatment. Similarly, lesion thickness in cranial regions reduced after +3 days, +5 days, and +11 days. In addition, the thickness was almost always less than 1 cm from +11 days after treatment. Jourquin et al. (2022) [35] found that about 80% of beef calves (>3 months of age) had complete reaeration of the lung at +3 days after florfenicol treatment. The results of our study appear to be similar considering that the threshold value of lung consolidation to perform the treatment was only 0.5 cm in the Jourquin et al. (2022) [35] study. On the contrary, a much greater thickness of lesion (4.5–5 cm) was evidenced at treatment day in our study. Lung consolidation identified by ultrasonography may represent an inflammatory process [36]. The use of anti-inflammatory drugs has been associated with a lower degree of lung injury probably due to the reduction in tissue inflammation [26,29]. Consequently, it is possible that the early treatment effect (reduced LLS and total area of lung consolidation at +1 day) may be due to a reduction in lung inflammation following meloxicam administration.

As previously discussed, the lung consolidations (air leakage into the lung tissue) may involve multiple types of lesions: viral pneumonias, bacterial pneumonias, chronic pneumonias, post-infection resolution, and scar tissue. Performing ultrasound monitoring increases the possibility of discerning between acute processes, both viral and bacterial, and chronic/inactive processes [1,3]. An assessment of lung lesion size has been proposed to distinguish between viral (<3 cm) and bacterial infections (≥3 cm) [3]. However, the study in [10] experimentally infected animals with bovine respiratory syncytial virus, and these animals presented both lesions between 2 and 4 cm and above 4 cm. Moreover, the distinction between viral and bacterial pneumonia turns out to be important for public health. Indeed, there is no curative therapy for viral infections, and antibiotics treatment is often used to control secondary bacterial infections [26,37]. Nevertheless, antibiotic-based metaphylactic treatments have provided highly variable or even no results [7,29]. Furthermore, mass medication is being discouraged in favor of individual parenteral treatments to prevent antimicrobial resistance through prudent use of antibiotics [11,38,39]. Therefore, ultrasonographic presentation of lung lesions could provide support in distinguishing between the two types of pneumonia and in treatment decision making. In particular, the presence of tubular structures with anechoic to partially echogenic content (mucopurulent exudate) represent fluid alveolograms/bronchograms [40]. Bacterial pneumonias are characterized by the presence of neutrophils within the bronchiolar or alveolar lumens, in contrast to viral pneumonias, which have the presence of mononuclear peri-bronchial infiltrates, with or without epithelial necrosis and lobular atelectasis [41]. The LLS score, although not yet validated, is based on the different presentation of lung lesions (i.e., liver-like or hepatization, fluid alveolograms/bronchograms) and could provide a valuable support for therapeutic choice in association with lesion size [1].

The pleural surface may be involved during BRD cases generating pleurisy. Pleurisy has been reported to be associated with bacterial infections, and it is distinguished into acute (fibrinous pleurisy associated with pleural effusion) and/or chronic (adhesive or dry pleurisy without fluid between pleurae) [23,37,40,42]. Pleural effusions show an increased echogenicity positively associated with cellularity, protein level, and presence of fibrin [43]. The presence of fibrinous pleuritis associated with pneumonia is reported to have a poor prognosis [40]. However, lung ultrasonography could be useful for monitoring the degree of effusion as response to drug therapy [43]. In our study, 28 animals of the TRT group displayed pleurisy with pleural effusions. The thickness of effusions decreased following treatment with florfenicol and meloxicam after +1 day to +5 days, and resolving in +7 days to +9 days for cranial regions and +3 days for middle regions. These results further suggested the importance of early diagnosis and treatment to succeed in increasing treatment efficacy.

Clinical scores and US of the last ante mortem evaluation presented fair accuracy when compared with necropsy examination (73–78%). Notably, clinical scores had a very low sensitivity (3.7–7.4%), indicating the high risk of false negatives (animals with lower respiratory tract affected and not identified and treated). The sensitivity found in our study was lower than normally reported (31–62%) [3]. However, this sensitivity was generally evaluated against lung ultrasonography with the risk of classification bias, i.e., when the comparator test is not a 100% accurate gold standard (necropsy examination) [3,10]. Furthermore, good diagnostic accuracy of clinical signs is reported when it is performed within 96 h of the BRD onset to decline thereafter [44]. In contrast, the last clinical and ultrasonographic evaluation of the animals was used for the comparison in this study and it was performed 3 to 7 days before slaughter (116 days after the last enrollment in TRT group and 94 days after the last BRD case). Regarding the US, it showed higher sensitivity than clinical scores (48% vs. 4–7%) and slightly lower specificity (90% vs. 100%). However, the US presented reduced sensitivity compared with the literature (48% vs. 77–94%) [3]. This result could be due to multiple effects. Indeed, lung ultrasound was performed at the end of the production cycle as opposed to evaluation performed within 12 weeks of life with euthanasia immediately following the ultrasound scan [41]. In addition, US was evaluated on calves and not on animals of at least 250 kg [9]. Lastly, the presence of pleural adhesions indicative of pleurisy and not present in the US score may have further reduced its sensitivity. However, it is necessary to consider that although lung ultrasonography provides a better diagnosis of lower respiratory tract infection compared to clinical signs, it is only a proxy for the gold standard (necropsy examination) [6]. Ante mortem ultrasound evaluations were found to be concordant with necropsy evaluations by 3–59% [10,45]. In contrast to the other tests, the LLS presented the highest accuracy (93%) with high sensitivity (85%) and specificity (96%). This result could be due to the discernment of the types of lung lesions identified through the LLS that are not grouped exclusively in lung consolidation.

## 5. Conclusions

In this study that monitored veal calf health during a complete production cycle, a majority of BRD cases occurred between 3 and 28 days after a calf’s arrival. The weekly lung scan of calves coupled with clinical examination led to early detection of BRD clinical cases, 5 days before most frequently used clinical scoring tools. Prompt treatment of calves with BRD with a fixed combination of florfenicol and meloxicam was associated with a high success rate, an absence of relapse, a rapid healing of lung lesions, and a similar growth and carcass quality to untreated healthy calves. In conclusion, targeted treatments following periodic clinical and ultrasonographic monitoring allowed an early diagnosis of active bronchopneumonia with relevant reduction in the prevalence of lung lesions at slaughter.

## Figures and Tables

**Table 1:** Average values (n. of diseased animals; diseased %) of clinical scores (Wisconsin and California), ultrasonography score (US), lung lesion score (LLS), and total lung consolidation area (cm^2^) between treated (TRT, n = 36) and control (CTR, n = 48) groups. For US and clinical scores, median, 1–3 interquartile range, min–max values were also provided.

Parameter	Group	Arrival	Treatment Day	End of the Cycle	SEM	*p*-Values
Wisconsin score	CTR	0.25 ^y ^ (1; 2.1%) (0; 0–0; 0–4)	0.68 ^b,x ^ (3; 6.3%) (0; 0–2; 0–4)	0.36 ^y ^ (0; 0%) (0; 0–0; 0–2)	0.10	0.007
	TRT	0.43 ^y ^ (0; 0%) (0; 0–1; 0–2)	2.05 ^a,x ^ (5; 13.8%) (2; 0.75–3; 0–7)	0.32 ^y ^ (0; 0%) (0; 0–0; 0–3)	0.20	
California score	CTR	0.64 ^xy ^ (3; 6.3%) (0; 0–0; 0–6)	0.77 ^b,x ^ (4; 8.3%) (0; 0–2; 0–9)	0.39 ^y ^ (0; 0%) (0; 0–0; 0–4)	0.13	<0.001
	TRT	0.76 ^y ^ (1; 2.7%) (0; 0–2; 0–5)	2.79 ^a,x ^ (9; 25%) (2; 1.5–4.25; 0–9)	0.46 ^y ^ (2; 5.6%) (0; 0–0; 0–6)	0.29	
US score	CTR	0.97 ^y ^ (0; 0%) (1; 0–1; 0–2)	1.09 ^b,xy ^ (0; 0%) (1; 0–1; 0–2)	1.31 ^b,x ^ (11; 22.9%) (1; 1–2; 0–5)	0.10	<0.001
	TRT	0.85 ^z ^ (0; 0%) (1; 0–1; 0–2)	4.69 ^a,x ^ (36; 100%) (4; 4–5; 3–5)	2.29 ^a,y ^ (8; 22.2%) (2; 1–2; 0–5)	0.25	
LLS score	CTR	1.11 ^z ^ (0; 0%)	2.00 ^b,y ^ (6; 12.5%)	5.93 ^b,x ^ (11; 22.9)	0.39	<0.001
	TRT	1.44 ^z ^ (0; 0%)	15.6 ^a, x ^ (30; 83.3%)	9.99 ^a, y ^ (11; 30.5%)	1.24	
Total lung consolidation area	CTR	1.53 ^y^	1.25 ^b,y^	3.68 ^x^	0.53	<0.001
	TRT	0.66 ^y^	30.1 ^a,x^	5.50 ^y^	2.56	

^a,b^ indicated significant differences between group; ^x–z^ indicated significant differences over time.

**Table 2:** Average daily gain (kg/day), live weight (kg) at the end of production cycle, cold carcass weight (kg), and carcass quality according to the S-EUROP classification in the treated (TRT, n = 33) and control (CTR, n = 48) groups.

Groups	ADG (kg/day)	Live Weight (kg)	Cold Carcass Weight (kg)	VE-2	VE-3	VU-2	VU-3	VR-2
CTR	1.50	316	181.6	47.9%	2.1%	41.7%	0.0%	8.3%
TRT	1.44	310	178.3	36.4%	0.0%	48.5%	3.0%	12.1%
SEM	0.04	6.69	3.81	/	/	/	/	/
*p*-value	0.406	0.521	0.557	0.308	0.405	0.548	0.230	0.575

**Table 3:** Consolidation areas (cm^2^) and thickness (cm) in the cranial regions of right and left lungs.

Follow-Ups	Consolidation of Cranial Regions			
	Area: Right Region	Thickness: Right Region	Area: Left Region	Thickness: Left Region
−7 days	0.48 ^d^	0.18 ^e^	0.04 ^d^	0.32 ^e^
−3 days	3.66 ^cd^	1.07 ^d^	3.38 ^bcd^	0.76 ^cde^
Day of diagnosis and treatment	13.07 ^a^	5.00 ^a^	15.63 ^a^	4.51 ^a^
+1 day	6.79 ^b^	4.06 ^ab^	6.66 ^b^	4.35 ^a^
+3 days	5.71 ^b^	3.91 ^b^	4.42 ^bc^	2.48 ^b^
+5 days	3.97 ^bc^	2.06 ^c^	2.31 ^cd^	1.26 ^c^
+7 days	2.95 ^bcd^	1.47 ^cd^	2.28 ^cd^	1.05 ^cd^
+9 days	1.11 ^d^	1.26 ^cd^	1.67 ^cd^	1.13 ^cd^
+11 days	1.01 ^d^	0.94 ^de^	1.47 ^cd^	0.67 ^cde^
+14 days	1.33 ^d^	0.96 ^de^	0.30 ^cd^	0.81 ^cde^
+21 days	1.48 ^d^	1.20 ^cd^	0.31 ^cd^	0.86 ^cde^
+28 days	2.17 ^cd^	1.55 ^cd^	0.35 ^cd^	0.89 ^cde^
+35 days	1.36 ^d^	1.00 ^d^	0.25 ^d^	0.72 ^cde^
+42 days	1.36 ^d^	0.79 ^de^	0.23 ^d^	0.65 ^de^
+49 days	1.39 ^d^	0.87 ^de^	0.24 ^d^	0.70 ^cde^
+56 days	1.81 ^cd^	0.93 ^de^	0.24 ^d^	0.61 ^de^
+63 days	1.69 ^cd^	1.08 ^d^	0.30 ^cd^	0.75 ^cde^
+70 days	1.67 ^cd^	1.02 ^d^	0.33 ^cd^	0.78 ^cde^
+77 days	1.75 ^cd^	1.08 ^d^	3.21 ^bcd^	0.96 ^cd^
+84 days	1.29 ^d^	0.66 ^de^	0.31 ^cd^	0.69 ^cde^
+91 days	1.51 ^d^	0.80 ^de^	2.46 ^bcd^	0.64 ^de^
+98 days	1.70 ^cd^	0.99 ^de^	3.01 ^bcd^	0.88 ^cde^
+105 days	1.57 ^cd^	0.82 ^de^	0.28 ^d^	0.87 ^cde^
+112 days	1.18 ^d^	0.50 ^de^	1.08 ^cd^	0.41 ^e^
+119 days	1.81 ^cd^	1.23 ^d^	1.26 ^cd^	0.70 ^de^
+126 days	1.13 ^d^	0.60 ^de^	1.06 ^cd^	0.32 ^e^
+133 days	1.32 ^d^	1.09 ^d^	2.18 ^cd^	0.83 ^cde^
+140 days	1.10 ^d^	0.50 ^de^	1.05 ^cd^	0.30 ^e^
+147 days	1.88 ^cd^	0.31 ^de^	0.34 ^cd^	0.10 ^e^
+154 days	1.50 ^d^	1.18 ^d^	2.58 ^bcd^	0.85 ^cde^
+161 days	2.52 ^cd^	0.69 ^de^	2.55 ^bcd^	0.68 ^de^
+168 days	2.54 ^cd^	1.01 ^d^	2.09 ^cd^	1.11 ^cd^
SEM	1.72	0.34	1.81	0.32
*p*-value	<0.001	<0.001	<0.001	<0.001

^a–e^ Significant differences over time.

**Table 4:** Pleural effusion thickness (cm) of the cranial and middle regions of both right and left lungs.

Follow-Ups	Cranial Right Region	Middle Right Region	Cranial Left Region	Middle Left Region
−7 days	0.00 ^c^	0.00 ^c^	0.00 ^b^	0.00 ^b^
−3 days	0.03 ^c^	0.04 ^b^	0.00 ^b^	0.00 ^b^
Day of diagnosis and treatment	0.22 ^ab^	0.07 ^a^	0.18 ^ab^	0.00 ^b^
+1 day	0.26 ^a^	0.02 ^bc^	0.24 ^a^	0.04 ^a^
+3 days	0.18 ^b^	0.00 ^c^	0.18 ^ab^	0.00 ^b^
+5 days	0.05 ^c^	0.00 ^c^	0.03 ^b^	0.00 ^b^
+7 days	0.00 ^c^	0.00 ^c^	0.01 ^b^	0.00 ^b^
+9 days	0.00 ^c^	0.00 ^c^	0.00 ^b^	0.00 ^b^
SEM	0.03	0.01	0.03	0.01
*p*-value	0.027	<0.001	0.039	0.009

^a–c^ Significant differences over time.

**Table 5:** Diagnostic test evaluations using the Wisconsin score, California score, ultrasonography score (US), and lung lesion score (LLS) at 3 to 7 days before slaughtering compared to the necropsy findings.

Statistic	Wisconsin Score		California Score	
	Value	95% CI	Value	95% CI
Accuracy	72.9%	62.9% to 81.5%	74.0%	64.0% to 82.4%
Sensitivity	3.70%	0.09% to 19.0%	7.41%	0.91% to 24.3%
Specificity	100%	94.8% to 100%	100%	94.8% to 100%
Positive Likelihood Ratio	\	\	\	\
Negative Likelihood Ratio	0.96	0.89 to 1.04	0.93	0.83 to 1.03
Positive Predictive Value	100%	2.50% to 100%	100%	15.8% to 100%
Negative Predictive Value	72.6%	71.1% to 74.1%	73.4%	71.3% to 75.4%
**Statistic**	**US**		**LLS**	
	**Value**	**95% CI**	**Value**	**95% CI**
Accuracy	78.1%	68.5% to 85.9%	92.7%	85.6% to 97.0%
Sensitivity	48.2%	28.7% to 68.1%	85.2%	66.3% to 95.8%
Specificity	89.9%	80.2% to 95.8%	95.7%	87.8% to 99.1%
Positive Likelihood Ratio	4.75	2.12 to 10.60	19.59	6.41 to 59.92
Negative Likelihood Ratio	0.58	0.40 to 0.84	0.15	0.06 to 0.38
Positive Predictive Value	65.0%	45.4% to 80.6%	88.5%	71.5% to 95.9%
Negative Predictive Value	81.6%	75.3% to 86.5%	94.3%	87.0% to 97.6%

## Data Availability

The data are available by sending an email to the corresponding author.

## Supplementary Materials

The following supporting information can be downloaded at: https://www.mdpi.com/article/10.3390/ani14233499/s1, Table S1: Clinical score according to the California system and the Wisconsin system; Table S2: Wisconsin score, California score, ultrasonography score (US), lung lesion score (LLS), and total area of lung consolidation (cm^2^) over time in the Treated Group (n = 36); Table S3: Consolidation areas (cm^2^) and thickness (cm) in the middle regions of right and left lungs.
