# Supplementary material for: Bovine Respiratory Disease in Veal Calves: Benefits Associated with Its Early Detection by Lung Ultrasonography and Its Prompt Treatment with a Single Dose of a Fixed Combination of Florfenicol and Meloxicam
Source: Animals (Basel). 2024 Dec 4;14(23):3499. doi: 10.3390/ani14233499 (PMC11640095; doi:10.3390/ani14233499)
Supplement: Supplementary file 1 [file animals-14-03499-s001.zip › animals-3285154-supplementary.pdf]

animals

MDPI

Supplementary Tables

Table S1. Clinical score according to the California system and the Wisconsin system.

California clinical score

Parameter	Normal condition	Abnormal condition
Nasal discharge	0	4
Ocular discharge	0	2
Ear droop or head tilt	0	5
Cough	0 (absent)	2 (present)
Breathing	0 (normal)	2 (rapid or difficult)
Rectal temperature	0 (<39.2 °C)	2 (≥39.2 °C)

Wisconsin clinical score

Parameter	Score 0	Score 1	Score 2	Score 3
Nasal discharge	Normal, serous discharge	Small amount of unilateral, cloudy discharge	Bilateral, cloudy, or excessive mucus	Copious, bilateral mucopurulent nasal discharge
Ocular discharge and ear position	Normal eye and ear	Mild ocular discharge or ear flicking	Moderate bilateral ocular discharge or slight unilateral ear drop	Heavy ocular discharge, severe head tilt, or bilateral ear droop
Cough score	No cough	Induce single	Induce repeated coughs or occasional spontaneous	Repeated spontaneous coughing
Rectal temperature	37.8-38.3 °C	38.4-38.8 °C	38.9-39.4 °C	>39.4 °C

Table S2. Wisconsin score, California score, ultrasonography score (US), lung lesion score (LLS), and total area of lung consolidation (cm^2) over time in the Treated Group (n=36). Data were expressed ad mean (median; 1 – 3 quartiles; minimum – maximum values).

Follow-ups	Wisconsin score	California score	US score	LLS score	Total lung consolidation
− 7 days	0.52 ^{fg} (0; 0-2; 0-2)	0.77 ^{efg} (0; 0-2; 0-5)	0.90 ^{g} (1; 0-1; 0-2)	1.30 ^{f}	6.54 ^{cd}
− 3 days	0.67 ^{fg} (0; 0-2; 0-2)	0.79 ^{efg} (0; 0-2; 0-4)	1.51 ^{f} (2; 1-2; 0-2)	4.02 ^{de}	8.68 ^{c}
Day of diagnosis and treatment	2.05 ^{cd} (2; 0.25-3; 0-4)	2.79 ^{cd} (2; 1.5-4.25; 0-9)	4.69 ^{a} (4; 4-5; 3-5)	15.56 ^{a}	30.06 ^{a}
+ 1 day	2.03 ^{d} (2; 2-3; 0-6)	2.75 ^{d} (2; 0.5-4.75; 0-9)	4.21 ^{ab} (4; 3-4; 2-5)	12.52 ^{b}	15.56 ^{b}
+ 3 days	2.77 ^{bc} (3; 3-5; 2-7)	3.80 ^{bc} (4; 2-6; 0-11)	3.64 ^{b} (3; 3-3; 2-5)	10.92 ^{b}	13.61 ^{b}
+ 5 days	4.47 ^{a} (4; 4-5; 0-6)	5.69 ^{a} (6; 6-8; 2-9)	2.41 ^{c} (2; 2-3; 1-4)	6.98 ^{c}	8.57 ^{c}
+ 7 days	3.34 ^{ab} (3; 2.5-4; 0-6)	4.07 ^{b} (4; 2-6; 0-11)	2.10 ^{cd} (2; 2-3; 1-4)	4.33 ^{de}	7.43 ^{c}
+ 9 days	1.66 ^{de} (2; 0-2; 0-5)	2.11 ^{d} (2; 0-3; 0-7)	1.90 ^{cdef} (2; 1-2; 1-4)	3.56 ^{de}	3.26 ^{e}
+ 11 days	0.87 ^{fg} (0; 0-2; 0-3)	0.85 ^{efg} (0; 0-2; 0-4)	1.68 ^{def} (1.5; 1-2; 0-2)	3.74 ^{de}	3.67 ^{e}
+ 14 days	0.63 ^{fg} (0; 0-2; 0-2)	0.62 ^{efg} (0; 0-2; 0-4)	1.69 ^{def} (1; 1-2; 0-4)	3.36 ^{e}	3.36 ^{e}
+ 21 days	0.85 ^{fg} (0; 0-2; 0-3)	0.90 ^{efg} (0; 0-2; 0-7)	1.75 ^{def} (1; 1-2; 0-4)	4.60 ^{de}	3.70 ^{de}
+ 28 days	0.80 ^{fg} (0; 0-2; 0-4)	0.82 ^{efg} (0; 0-2; 0-4)	1.82 ^{def} (1; 1-2; 0-5)	4.39 ^{de}	3.11 ^{e}
+ 35 days	0.85 ^{fg} (0; 0-2; 0-4)	1.09 ^{e} (0; 0-2; 0-6)	1.70 ^{def} (1; 1-2; 0-5)	3.30 ^{e}	3.95 ^{de}
+ 42 days	0.78 ^{fg} (0; 0-2; 0-3)	0.81 ^{efg} (0; 0-2; 0-4)	1.74 ^{def} (1; 1-2; 1-5)	4.09 ^{de}	3.27 ^{e}
+ 49 days	0.63 ^{fg} (0; 0-2; 0-2)	0.70 ^{efg} (0; 0-2; 0-4)	1.60 ^{ef} (1; 1-2; 0-5)	3.36 ^{e}	3.56 ^{e}
+ 56 days	0.75 ^{fg} (0; 0-2; 0-4)	0.88 ^{efg} (0; 0-2; 0-6)	1.61 ^{ef} (1; 1-2; 0-5)	3.75 ^{de}	3.73 ^{de}
+ 63 days	0.48 ^{fg} (0; 0-1.5; 0-6)	0.49 ^{fg} (0; 0-1.5; 0-8)	1.82 ^{def} (2; 1-2; 0-5)	3.54 ^{de}	3.31 ^{e}
+ 70 days	1.05 ^{ef} (1; 0-2; 0-3)	0.99 ^{efg} (1; 0-2; 0-4)	1.89 ^{def} (2; 1-2; 0-5)	3.92 ^{de}	3.94 ^{de}
+ 77 days	0.97 ^{fg} (0; 0-2; 0-3)	1.02 ^{ef} (1; 0-2; 0-6)	1.82 ^{def} (2; 1-2; 0-5)	4.35 ^{de}	3.28 ^{e}
+ 84 days	0.61 ^{fg} (0; 0-2; 0-2)	0.63 ^{efg} (0; 0-2; 0-4)	1.64 ^{def} (2; 1-2; 0-5)	3.56 ^{de}	3.33 ^{e}
+ 91 days	0.85 ^{fg} (0; 0-2; 0-2)	0.85 ^{efg} (0; 0-2; 0-4)	1.51 ^{f} (1; 1-2; 0-5)	4.75 ^{cde}	3.28 ^{e}
+ 98 days	0.73 ^{fg} (0; 0-2; 0-2)	1.05 ^{ef} (1; 0-2; 0-6)	1.96 ^{cde} (2; 1-2; 1-5)	5.83 ^{cd}	2.78 ^{e}
+ 105 days	0.42 ^{g} (0; 0-0.5; 0-2)	0.48 ^{g} (0; 0-2; 0-4)	1.71 ^{def} (1; 1-2; 0-5)	2.89 ^{ef}	2.71 ^{e}
+112 days	0.37 ^{g} (0; 0-0; 0-2)	0.33 ^{g} (0; 0-0; 0-2)	1.79 ^{def} (2; 1-2; 0-3)	2.57 ^{ef}	2.49 ^{e}
+119 days	0.47 ^{fg} (0; 0-0; 0-2)	0.43 ^{g} (0; 0-0; 0-2)	1.39 ^{f} (1; 0-2; 0-5)	2.89 ^{ef}	5.39 ^{de}
+126 days	0.55 ^{fg} (0; 0-0.5; 0-2)	0.75 ^{efg} (0; 0-0.5; 0-5)	1.50 ^{f} (1; 1-1.25; 0-3)	2.08 ^{ef}	2.59 ^{e}
+133 days	0.53 ^{fg} (0; 0-0.5; 0-2)	0.50 ^{fg} (0; 0-0.5; 0-2)	1.36 ^{fg} (1; 0-1.25; 0-5)	2.42 ^{ef}	4.98 ^{de}
+140 days	0.22 ^{g} (0; 0-0.25; 0-1)	0.41 ^{g} (0; 0-0.5; 0-2)	1.12 ^{fg} (1; 0.75-1.25; 0-2)	2.94 ^{ef}	2.20 ^{e}
+147 days	0.30 ^{g} (0; 0-0; 0-2)	0.27 ^{g} (0; 0-0; 0-2)	1.19 ^{fg} (1; 1-1.5; 0-2)	2.30 ^{ef}	2.62 ^{e}
+154 days	0.34 ^{g} (0; 0-0; 0-4)	0.45 ^{g} (0; 0-0; 0-6)	1.18 ^{fg} (1; 1-2; 0-5)	2.09 ^{ef}	4.46 ^{de}
+161 days	0.13 ^{g} (0; 0-0; 0-2)	0.12 ^{g} (0; 0-0; 0-2)	1.15 ^{fg} (1; 1-2; 0-4)	2.47 ^{ef}	4.22 ^{de}
+168 days	0.27 ^{g} (0; 0-0; 0-2)	0.25 ^{g} (0; 0-0; 0-2)	1.58 ^{ef} (1; 1-4; 0-5)	3.49 ^{de}	5.46 ^{de}
SEM	0.27	0.35	0.20	1.0	2.22
p-value	<0.001	<0.001	<0.001	<0.001	<0.001

$^{a\text{-}g}$ Significant differences over time

Table S3. Consolidation areas (cm^2) and thickness (cm) in the middle regions of right and left lungs.

Follow-ups	Consolidation of Middle rgions			
	**Area – right region**	**Thickness – right region**	**Area – left region**	**Thickness – left region**
− 7 days	0.01 ^{b}	0.01	0.12 ^{b}	0.14
− 3 days	0.02 ^{b}	0.00	0.12 ^{b}	0.00
Day of diagnosis and treatment	0.38 ^{a}	0.32	0.75 ^{a}	0.60
+ 1 day	0.38 ^{a}	0.08	0.27 ^{b}	0.14
+ 3 days	0.39 ^{a}	0.24	0.11 ^{b}	0.11
+ 5 days	0.05 ^{b}	0.05	0.12 ^{b}	0.11
+ 7 days	0.01 ^{b}	0.01	0.14 ^{b}	0.10
+ 9 days	0.01 ^{b}	0.00	0.12 ^{b}	0.11
+ 11 days	0.01 ^{b}	0.00	0.12 ^{b}	0.15
+ 14 days	0.02 ^{b}	0.00	0.13 ^{b}	0.12
+ 21 days	0.03 ^{b}	0.01	0.13 ^{b}	0.09
+ 28 days	0.14 ^{ab}	0.07	0.13 ^{b}	0.11
+ 35 days	0.11 ^{ab}	0.07	0.21 ^{b}	0.13
+ 42 days	0.08 ^{b}	0.06	0.19 ^{b}	0.16
+ 49 days	0.04 ^{b}	0.06	0.22 ^{b}	0.09
+ 56 days	0.04 ^{b}	0.02	0.18 ^{b}	0.15
+ 63 days	0.03 ^{b}	0.02	0.25 ^{b}	0.16
+ 70 days	0.15 ^{ab}	0.10	0.49 ^{ab}	0.34
+ 77 days	0.14 ^{ab}	0.10	0.36 ^{b}	0.33
+ 84 days	0.14 ^{ab}	0.10	0.41 ^{b}	0.38
+ 91 days	0.13 ^{ab}	0.10	0.33 ^{b}	0.37
+ 98 days	0.18 ^{ab}	0.13	0.40 ^{b}	0.27
+ 105 days	0.18 ^{ab}	0.17	0.38 ^{b}	0.27
+112 days	0.01 ^{b}	0.01	0.28 ^{b}	0.15
+119 days	0.17 ^{ab}	0.13	0.21 ^{b}	0.19
+126 days	0.11 ^{ab}	0.01	0.19 ^{b}	0.14
+133 days	0.17 ^{ab}	0.12	0.13 ^{b}	0.12
+140 days	0.01 ^{b}	0.01	0.16 ^{b}	0.14
+147 days	0.01 ^{b}	0.01	0.13 ^{b}	0.16
+154 days	0.19 ^{ab}	0.01	0.17 ^{b}	0.19
+161 days	0.01 ^{b}	0.01	0.21 ^{b}	0.19
+168 days	0.15 ^{ab}	0.14	0.23 ^{b}	0.17
SEM	0.13	0.08	0.15	0.17
p-value	0.008	0.061	<0.001	0.064

$^{a-b}$ Significant differences over time
